# ABO-Incompatible Living Donor Liver Transplantation from Hepatitis B Core Antibody Positive Donor to Hepatitis C Liver Cirrhosis Recipient: A Case Report

**DOI:** 10.1155/2014/507621

**Published:** 2014-06-22

**Authors:** Akira Umemura, Hiroyuki Nitta, Akira Sasaki, Takeshi Takahara, Yasushi Hasegawa, Go Wakabayashi

**Affiliations:** Department of Surgery, Iwate Medical University, 19-1 Uchimaru, Morioka 020-8505, Japan

## Abstract

Herein, we describe an extremely rare experience of a patient with liver cirrhosis from hepatitis C virus (LC-HCV) who underwent an ABO-incompatible living donor liver transplantation (ABO-I-LDLT) using a hepatitis B core antibody (HBc-Ab) positive donor's liver graft. A 47-year-old Japanese woman with end stage LC-HCV, as a recipient, was preoperatively administered rituximab, mycophenolate mofetil, and steroids without plasma exchange. A routine ABO-I-LDLT procedure was applied using her daughter's HBc-Ab positive liver graft. Prophylaxis of the hepatitis B virus (HBV) infection using hepatitis B immunoglobulin (HBIG) and entecavir had been properly administered. Three months after the ABO-I-LDLT, HCV hepatitis relapsed. To date, this patient has been under antiviral therapy and prophylaxis of HBV infection using HBIG, while entecavir has been continued. The cognitions and techniques with regard to ABO-I-LDLT, prophylaxis of HBV cross infection, various patterns of immunosuppression, and antiviral therapy for HCV relapse are indispensable in managing a transplant recipient. According to the prophylaxis of HBV cross infection under ABO-I-LDLT, it may be very important to keep the HBs-Ab titer higher than usual for HBV naïve recipients, because severe systemic immunosuppression can cause *de novo* hepatitis.

## 1. Introduction

Liver cirrhosis by hepatitis C virus (LC-HCV) is a common indication for living donor liver transplantation (LDLT), with the advancement of surgical procedures and antiviral therapies [1]. According to the hepatitis B core antibody (HBc-Ab) positive donor in LDLT, liver grafts can be safely used for hepatitis B surface antigen (HBs-Ag) negative recipients under the administration of prophylaxis using hepatitis B immunoglobulin (HBIG) and entecavir [2]. However, although ABO-incompatible LDLT (ABO-I-LDLT) in adults has not exhibited satisfactory graft survival rates and incidences of complications [3], the employment of plasma exchange (PE), portal vein infusion therapy (PVIT), and immunosuppressive treatment targeted to B cells using rituximab has dramatically improved the outcome [4–6].

In Japan, however, the number of deceased donor liver transplantations (DDLT) has gradually increased since the enforcement of a new law on organ transplantation in 2010, but LDLT is still the most frequent treatment option for patients with end-stage liver disease because of the organ shortage [4, 7]. Patients with end-stage liver disease commonly give up if living donors are not available, and given this unique social context in Japan, we describe our extremely rare experience with an LC-HCV patient who underwent ABO-I-LDLT using an HBc-Ab positive donor's liver graft.

## 2. Case Report

A 47-year-old Japanese woman with a history of transfusion during labor was diagnosed with LC-HCV and administered liver supporting therapy for several years. After noticing a distension of her abdomen and icteric skin, she was referred to our clinic to consider LDLT. Physical examination revealed the accumulation of ascites, jaundice, and edema of her lower extremities. Routine laboratory investigations showed a serum total bilirubin (T-Bil) of 4.6 mg/dL, aspartate aminotransferase (AST) of 50 IU/L, alanine aminotransferase (ALT) of 35 IU/L, albumin of 3.3 g/dL, a prothrombin time-international normalized ratio (PT-INR) of 1.36, and platelet count of 3.7 × 10^4^/*μ*L and her initial Child-Pugh and model for end-stage liver disease scores were 11 and 16, respectively.

This patient's viral marker status is summarized in Table 1. These data revealed that she had not been previously exposed to hepatitis B virus (HBV). After her admission, we attempted to choose a living donor candidate among her relatives; however, there was no living donor candidate who could meet the general selection criteria of our institution.

A 22-year-old daughter of the patient who underwent a living donor workup was excluded as a donor candidate due to her ABO incompatibility and prior infection with HBV. However, there were no other patient matches that were living donors. Our procedure of ABO-I-LDLT and clinical research of this case have been approved by our institutional ethics committee. After a detailed explanation and informed consent, the patient agreed to undergo ABO-I-LDLT using an HBc-Ab positive liver graft, and her daughter was used as a living donor.

According to the preoperative estimation of the donor, routine laboratory investigations showed only normal liver function; the donor's blood type and viral marker status are summarized in Table 1. In addition, liver imaging studies using enhanced and drip infusion cholecystocholangiography computed tomography (CT) showed no significant anatomical variations or abnormalities, and the preoperative liver needle biopsy did not show any noticeable pathological changes. The right lobe volume of the donor was estimated to be 59.4% of the whole liver volume on CT volumetry, while the graft volume (GV)/recipient body weight and GV/standard liver volume were 0.86% and 51.5%, respectively (Table 2). For these reasons, a right lobe graft was chosen for transplantation into the patient.

Two weeks before the operation, 375 mg/m^2^ of rituximab (anti-CD20 antibody) was administered to the recipient. Therefore, the CD19- and CD20-positive B cell counts promptly descended under sensitivity. As the isoagglutinin titers of anti-A and anti-B were 32 × and 16 ×, respectively, preoperative PE was not performed. This patient was also treated with 500 mg of mycophenolate mofetil (MMF) and 5 mg of prednisolone for one week until LDLT.

The routine LDLT procedure was applied for both the donor and the recipient. An intraoperative liver wedge biopsy of the donor revealed no evidence of steatosis, cholecystitis, or overt HBV infection. Immediately following total hepatectomy and splenectomy, 20,000 IU of HBIG was infused into the recipient. A double-lumen catheter was inserted through the portal vein via the ileocolic vein to employ PVIT after vessel and bile duct reconstructions.

**Figure 1:**
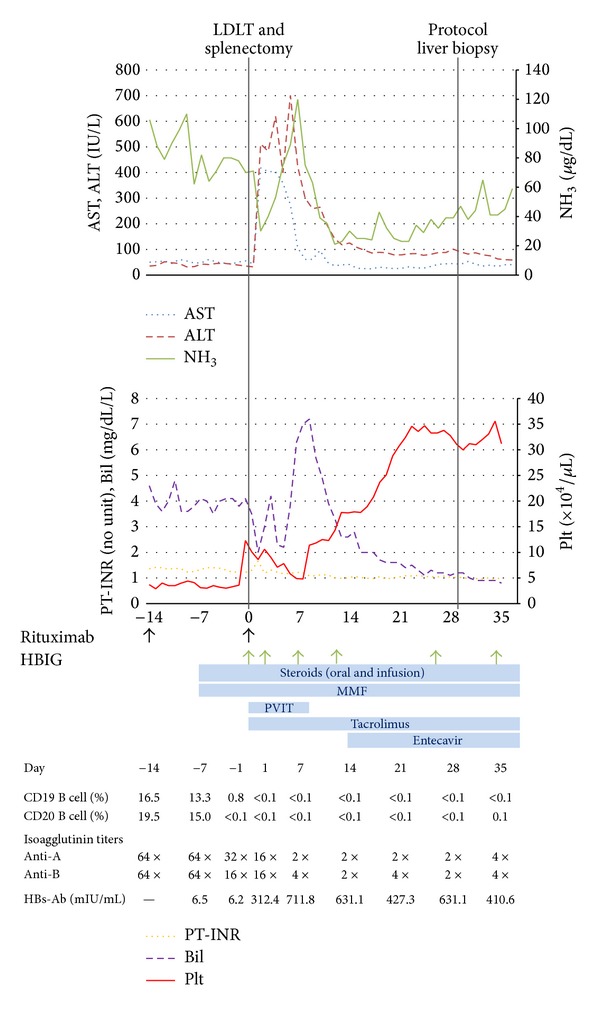
Major treatments, events, and laboratory results in the clinical course. Panel shows a timeline of the recipient's clinical course until discharge, with arrows indicating points of administration of rituximab and HBIG. Periods of steroids, MMF, PVIT, tacrolimus, and entecavir are indicated by the gray bars. Results of CD19- and CD20-positive B cell count, isoagglutinin titers of anti-A and anti-B, and HBs-Ab are shown below the graph.

Perioperative data and treatments of the recipient are shown in Figure 1. Following LDLT, an additional 375 mg/m^2^ of rituximab was administered to the recipient, and steroids, MMF, and tacrolimus were administered. PVIT was also employed for 7 days using prostaglandin E1 and gabexate mesilate. In addition to the routine postoperative treatment, the CD19- and CD20-positive B cell counts, isoagglutinin titers of anti-A and anti-B, and HBs-Ab were frequently monitored. According to prophylaxis of the HBV cross infection, 2000 IU of HBIG was properly administered so that the HBs-Ab was over 300 mIU/mL, and 0.5 mg of entecavir (guanosine analogue) was also added starting from 2 weeks after the LDLT.

Sudden elevations in the hepatic enzymes were observed around the 7th postoperative day, without any impeded blood flow, including outflow block; therefore, acute rejection was highly suspected. As temporal dose increments of steroid and MMF were appropriately administered, liver function gradually improved. The patient recovered progressively without any other complications, and the protocol liver biopsy on postoperative day 30 revealed no evidence of rejection, HCV relapse, and HBV infection. She was discharged from the hospital on postoperative day 39.

During the outpatient followup, MMF and tacrolimus were converted to mizoribine and cyclosporine from the viewpoint of the inhibition of HCV ribonucleic acid (HCV-RNA) replication and possibility of administration of pegylated interferon-*α* 2a (PEG-IFN-*α* 2a) therapy [8, 9]. She was followed every two weeks and administered liver biopsies every month to monitor for HCV relapse due to severe immunosuppression for ABO-I-LDLT. Three months after the ABO-I-LDLT, routine laboratory investigations and liver biopsy specimens showed HCV relapse. The hepatitis C virus core antigen and HCV-RNA at that time were 79,107.2 fmol/L and 7.8 Log IU/mL, respectively. The patient was promptly started on PEG-IFN-*α* 2a therapy with ribavirin and prednisolone for immunosuppression, which was gradually tapered. To date, she has been under antiviral therapy and prophylaxis of HBV infection using HBIG, and the entecavir has been continued.

## 3. Discussion

Although an ABO-I-LDLT was conducted, the HBc-Ab positive donor's liver graft and antiviral therapy for HCV relapse after LDLT are very important topics for transplant surgeons, since these problems have not been completely resolved to date [1–6]. With regard to DDLT in Japan, ABO incompatibility and the possibility of HBV cross infection are strictly considered when the Japan Organ Transplant Network determines the recipient for rare deceased liver grafts. However, with regard to LDLT, when these factors are duplicated between the recipient and the living donor, transplant surgeons must be well acquainted with treatments of ABO-I-LDLT, prophylaxis of HBV cross infection, and antiviral therapy for HCV relapse. We were unable to find any reports about ABO-I-LDLT from an HBc-Ab positive donor to an HCV recipient in the literature.

With regard to ABO-I-LDLT, Tanabe et al. [4, 5] and Egawa et al. [6] reported that triplet treatment consisting of PE, PVIT, and rituximab has dramatically improved the outcome of ABO-I-LDLT. In addition, the recent development of immunosuppression, especially antimetabolites and calcineurin inhibitors, has markedly decreased antibody-mediated rejection [3, 4, 10]. In this case, since the CD19- and CD20-positive B cell counts and isoagglutinin titers of anti-A and anti-B were very low before ABO-I-LDLT, a perioperative PE was never performed.

One of the current efforts to overcome the organ shortage in Japan is based on the use of liver grafts that are from living donors that are HBc-Ab positive [2]. However, the liver grafts from HBc-Ab positive donors are currently main sources of* de novo* hepatitis due to HBV cross infection after LDLT; therefore, HBs-Ag negative recipients should receive appropriate prophylaxis [2, 11–13]. Although the combination of HBIG and lamivudine or entecavir is often used [11, 12], Chotiyaputta et al. [13] reported that entecavir monotherapy without HBIG is sufficient in preventing HBV cross infection in HBs-Ag negative recipients of HBc-Ab positive donor liver grafts. In this case, not entecavir monotherapy but a combination of HBIG infusion and entecavir was administered because the risk of HBV cross infection was thought to be higher from the viewpoint of severe immunosuppression accompanied by ABO-I-LDLT. In addition, the HBs-Ab titer was also kept higher than usual. However, to correctly answer this question, a well-planned study should be performed to definitively determine the nature of the relationship between these prophylactic agents and incidences of* de novo* hepatitis due to HBV cross infection in ABO-I-LDLT from HBc-Ab positive living donors to HBs-Ag negative recipients.

## 4. Conclusion

We herein described our extremely rare experience with an LC-HCV patient who underwent ABO-I-LDLT using an HBc-Ab positive donor's liver graft. In such a case, in addition to the routine LDLT procedure and postoperative management, cognitions, and techniques about ABO-I-LDLT, the prophylaxis of HBV cross infection, various patterns of immunosuppressive drugs, and antiviral therapy for HCV relapse are indispensable in the management of the recipient. Particularly according to prophylaxis of HBV cross infection under ABO-I-LDLT, it may be very important to keep the HBs-Ab titer higher than usual for HBV naïve recipients, because severe systemic immunosuppression can cause* de novo* hepatitis.

## Figures and Tables

**Table 1:** Preoperative blood type, HBV and HCV marker status of the recipient and the living donor.

	Recipient	Donor
Blood type		
	O Rh (+)	A Rh (+)

Viral marker		
HBs-Ag (IU/mL)	<0.05	0.1
HBs-Ab (mIU/mL)	6.5	859.7
HBc-Ab (S/CO)	<1.0	99.9
HBe-Ag (S/CO)	<0.5	0.1
HBe-Ab (%)	<35	>100
HCV-Ab (COI)	98.4	0.1
HCV-CA (fmol/L)	14,569.2	—
HCV-RNA (log⁡IU/mL)	6.7	—
HCV genotype	1b	—

Abbreviations: HBs-Ag, hepatitis B surface antigen; HBs-Ab, hepatitis B surface antibody; HBc-Ag, hepatitis B core antigen; HBc-Ab, hepatitis B core antibody; HBe-Ag, hepatitis B envelope antigen; HBe-Ab, hepatitis B envelope antibody; HCV-Ab, hepatitis C virus antibody; HCV-CA, hepatitis C virus core antigen, HCV-RNA, hepatitis C virus ribonucleic acid.

**Table 2:** Preoperative estimations of the recipient's standard liver volume and donor's graft volume.

	Recipient	Donor
Physical findings		
Height (cm)	163	166
Body weight (kg)	79.8	59.0
BSA (m^2^)	1.87	1.66
SLV (mL)	1325	

CT volumetry		
WLV (mL)		1150
Right lobe (mL)		683
GV/WLV (%)		59.4
GV/RBW (%)		0.86
GV/SLV (%)		51.5

Abbreviations: BSA, body surface area; SLV, standard liver volume; CT, computed tomography; WLV, whole liver volume; GV, graft volume; RBW, recipient body weight.
